# Efficiency and Resource Allocation in Government Hospitals in Saudi Arabi: A Casemix Index Approach

**DOI:** 10.3390/healthcare11182513

**Published:** 2023-09-11

**Authors:** Abdulrahman Alshehri, Bander Balkhi, Ghada Gleeson, Ehab Atassi

**Affiliations:** 1 National Casemix Center of Excellence, Riyadh 13315, Saudi Arabia; aalshehri@phap.sa (A.A.); gglesson@phap.sa (G.G.); eatassi@phap.sa (E.A.); 2 Department of Clinical Pharmacy, College of Pharmacy, King Saud University, Riyadh 11451, Saudi Arabia

**Keywords:** healthcare system, case mix index, efficiency, utilization, spending, resource allocation

## Abstract

In Saudi Arabia, the evaluation of healthcare institutions’ performance and efficiency is gaining prominence to ensure effective resource utilization. This study aims to assess the efficiency of government hospitals in Saudi Arabia using the case mix index (CMI) approach. Comprehensive data from 67 MoH hospitals were collected and analyzed. The CMI was calculated by assigning weights to different patient groups based on case complexity and resource requirements, facilitating comparisons of hospital performance in terms of resource utilization and patient outcomes. The findings reveal variations in the CMI across hospitals in relation to size and type. The average CMI was 1.26, with the highest recorded at 1.67 and the lowest at 1.02. Medical cities demonstrated the highest CMI (1.47), followed by specialized hospitals (1.32), and general hospitals (1.21). The study highlights opportunities for enhancing productivity and efficiency, particularly in hospitals with lower CMI, by benchmarking against peer institutions with similar capacities and patient case mix. These findings have significant implications for hospital operations and resource allocation policies, supporting ongoing efforts to improve the efficiency of government hospitals in Saudi Arabia. By incorporating these insights into healthcare strategies, policymakers can work towards enhancing the overall performance and effectiveness of the healthcare system.

## 1. Introduction

In the Kingdom of Saudi Arabia, healthcare demand is continuously growing due to several factors such as population growth, demographics changes, the prevalence of chronic diseases, and high premature mortality rates [1]. These factors lead to increased demand for healthcare, causing the government to allocate more resources to meet the population healthcare needs. Compared to many other countries, Saudi Arabia’s healthcare expenditure as a percentage of its gross domestic product (GDP) is relatively high. Therefore, transforming healthcare sector became a necessity to ensure its efficiency and sustainability, and for that it was one of the Saudi Vision 2030 main components. As a result, the Health Sector Transformation Program (HSTP) was established with the mission of restructuring healthcare to be comprehensive, effective, integrated, and value-based in delivering care to citizens, residents, and visitors [2].

Healthcare in Saudi Arabia is primarily provided to approximately 70% of the population through the public sector. This segment includes public sector workers who are covered by government-sponsored health insurance plans. These plans typically offer access to medical services provided by various public entities, including the Ministry of Health (MOH), Ministry of National Guard, Ministry of Defense, Ministry of Interior, and other government healthcare facilities. On the other hand, the remaining 30% of the population, consisting of both Saudi citizens and expatriates, seek healthcare services from private providers. To ensure access to healthcare, individuals in this segment are obligated to have private health insurance, which is typically provided by their employers [3].

The Ministry of Health (MOH) is considered the largest public provider of healthcare through its network, which includes about 250 hospitals of different levels and more than 2000 primary care centers that are distributed around the country. Before the implementation of HSTP, MOH was responsible for regulating, financing, and providing healthcare to the public. However, HSTP intended to separate these three roles of the ministry by establishing a new entity to be the unified national payor of healthcare and corporatizing providers through clustering them into 21 geographical clusters where they can provide care to their registered beneficiaries. This new model of care allows the clusters to be accountable for the health of their beneficiaries. Thus, it is crucial to understand how to utilize these resources in the most efficient and effective manner to achieve desired outcomes and address the evolving healthcare landscape.

Efficiency becomes a key consideration in the healthcare sector, as the scarcity of available resources pose challenges to deliver quality patient care while optimizing resource utilization. Efficiency in the healthcare sector refers to achieving system objectives with the resources available. It encompasses two types of efficiency: technical efficiency and allocative efficiency. Technical efficiency ensures effective use of resources within healthcare facilities, while allocative efficiency involves strategic resource allocation for better population health. Integrating both leads to improved patient care and sustainable healthcare systems [4,5,6,7] Policymakers are increasingly emphasizing the evaluation of healthcare systems and programs to ensure their efficiency and value for money [8,9]. Measuring efficiency enables policymakers to identify areas for improvement and cost reduction, ultimately leading to better resource allocation and enhanced healthcare outcomes. However, focusing solely on efficiency is not enough. Evidence-based decision making ensures that interventions achieve desired health outcomes effectively [10,11]. By integrating efficiency and effectiveness, healthcare systems can provide top-notch care while maximizing resource allocation for optimal patient outcomes. 

Hospitals serve as essential healthcare providers and account for a significant portion of total health expenditure. Therefore, optimizing hospital efficiency is a critical step in establishing more effective and sustainable health systems. Efficient hospitals can offer more effective treatments, reduce waiting times, and improve patient outcomes, ultimately contributing to the overall health and well-being of the population. Efficiency metrics like average length of stay (ALOS) have proven to be valuable in identifying inefficiencies in patient flow and resource utilization. By analyzing these metrics, hospital administrators can make informed decisions to optimize bed allocation, staffing, and scheduling, leading to improved patient turnover and reduced waiting times. Additionally, hospitals set efficiency targets to benchmark their performance with national or international standards. Comparing their efficiency with peers helps to identify areas of improvement and set enhancement plans for financial and operational performance [7]. Extensive research has shed light on the widespread technical inefficiencies present in hospitals across countries with varying economic development, leading to significant financial losses [12]. Moreover, optimizing hospital efficiency contributes to the overall sustainability of the healthcare system. As healthcare costs continue to rise, achieving better efficiency in hospitals can help in cost containment and resource optimization [13]. 

One notable area where inefficiencies may arise is the monitoring of hospitals and healthcare providers. Without an efficient mechanism in place, there can be alarming levels of waste and inefficiencies within the system. A study conducted in 2020 using DEA methodology assessed the technical efficiency of 91 MOH hospitals in Saudi Arabia and found that these hospitals were operating inefficiently, with an average efficiency score of 75%. This score represents the ratio of healthcare outputs and outcomes achieved relative to the inputs utilized [14]. This efficiency score implies that there is significant room for improvement in how resources are utilized and managed within these MOH hospitals. This suggests that there are inefficiencies in the delivery of healthcare services, leading to suboptimal outputs and outcomes relative to the resources invested. With healthcare transformation and the adoption of the new model of care based on the principle of value-based care, it is critical to establish mechanisms that ensure the efficient utilization of resources in healthcare delivery to achieve optimal outcomes. These outcomes should be highly relevant to beneficiaries in terms of quality and access to care, and specific measures should be selected to identify the problem of interest [15].

The public health system operates under limited economic resources, necessitating a thorough understanding of resource utilization and distribution. Previous studies have employed traditional methods such as data envelopment analysis (DEA) and stochastic frontier analysis (SFA) to assess resource efficiency [4,14,16]. Studies utilizing DEA have revealed significant variations in efficiency levels among hospitals, indicating potential area for improvement [6]. By identifying sources of inefficiency, healthcare providers and policymakers can implement targeted interventions to enhance resource utilization and streamline processes [17]. Similarly, studies employing SFA to examine the technical and allocative efficiency of hospitals demonstrated that ineffective resource utilization and suboptimal decision-making processes are contributors to inefficiencies in hospital operations [11]. However, when hospitals exhibit variation in case mix, these approaches may yield biased comparisons. To address this issue, researchers have recommended the utilization of hospital production data adjusted by diagnosis related groups (DRGs) weights to account for case mix differences [18]. This adjustment helps to account for differences in case complexity and ensures more accurate and fair comparisons between hospitals.

Case mix index (CMI) approach has gained recognition for evaluating efficiency and resource utilization. This approach takes into account the complexity of patient cases and provides a benchmark for assessing hospitals’ performance based on key performance indicators. Several key KPIs within the CMI domain serve as proxy measures for evaluating hospitals’ ability to handle complex cases and their productivity [19]. By considering patient diversity, clinical complexity, and resource utilization, CMI provides an indicator of treatment costs and enables fair comparisons between hospitals. Furthermore, CMI allows adjustments for the amount of funds a hospital get each year based on its patient case mix, which is the relative weights for all patients to give a hospital-specific base rate. CMI is a metric used to quantify the complexity and severity of patients treated in a hospital or healthcare facility. A higher CMI indicates that the hospital is treating more complex and resource-intensive cases, while a lower CMI suggests less complexity and resource utilization. It is important to note that there is no fixed target CMI, as it varies depending on the patient population and the specific context of the hospital. Hospitals with specialized services or high-acuity cases may have a higher CMI, while hospitals with more routine cases may have a lower CMI. The key is to use CMI as a tool for benchmarking and identifying areas for improvement in resource utilization and patient care.

The CMI approach has been successfully implemented in various countries to optimize funding allocation and enhance hospital efficiency. For example, in Australia, with the introduction of activity-based funding, incorporating the CMI led to improvements in cost-effectiveness and resource allocation [20]. In Canada, the CMI has been used to assess hospital performance and guide policy decisions regarding resource allocation [21]. The CMI approach offers a framework to assess efficiency and resource utilization, enabling informed decisions on funding based on patient case mix and complexity of care. This study aims to examine the efficiency of government hospitals in Saudi Arabia using the CMI and other metrics, providing valuable insights for optimizing resource allocation and enhancing overall efficiency. The findings will provide policymakers and healthcare administrators with evidence about the current state of hospital efficiency, resource utilization patterns, and patient complexity. This information will support evidence-based decision making, process improvement initiatives, and effective resource allocation, ultimately leading to enhanced quality of care and better outcomes for patients.

## 2. Materials and Methods

### 2.1. Study Design

A cross sectional retrospective-analytic study was conducted to assess the efficiency of various MOH hospitals for which complete data are available. The CMI is a statistical tool that can be used to assess the productivity and efficiency as well complexity of services. The efficiency of the hospital was measured using CMI with a focus on the following indicators:

- Technical efficiency: This indicator determines whether hospitals are utilizing their capacity effectively, allowing them to treat more patients with same capacity and resources. This eventually reduces the average cost per DRG. Assessing technical efficiency involves analyzing patients’ average length of stay (ALOS).
- Allocative efficiency: investigates whether the patients were treated in an appropriate clinical manner as it is perceived that healthcare provided in most cases could have been delivered in less intensive settings than the actual settings. To assess allocative efficiency, we will investigate the ratio of same-day cases or day cases and the number of days of surgery attendance (DOSA) before planned surgeries.
- Productivity of hospital: This indicator describes the volume of work undertaken by each cluster within a specific time period. Productivity of hospitals is not only measured by the number of patients or reported episodes, but also assessed by the total of weighted episodes.
- Complexity of care: This aspect describes the average degree of difficulty or relative cost of the work undertaken by each cluster.

### 2.2. Data Sources

This study was based on cross-sectional activity data of 67 MOH hospitals. The data collection was conducted using the ABM portal, a web-based data submission platform developed by the MOH. This platform was chosen due to its convenience and accessibility, making it the most suitable resource for conducting this analysis. Various variables including patients’ demographics, hospitals information, episode details, DRG, MDC (major diagnostic category), DRG weight, and procedures were collected from MOH hospitals in order to compare the efficiency across various hospitals and clusters. These variables were extracted from the hospital activity data for inpatient services in 2019 from 67 public hospitals and medical cities. These hospitals are distributed across 21 clusters and provide acute care for both medical and surgical conditions. Hospitals included in the analysis were categorized in two different ways, with the first categorization based on MOH classification of general hospitals, specialized hospitals, and medical cities (MC), and the second categorization based on Gok and Sezen’s categorization: small (less than 200 beds), lower-medium (200–299 beds), upper-medium (300–499 beds), and large (500 or more beds) hospitals (Table 1). CMI was calculated by dividing the sum of all episodes’ DRG-relative weights by the total number of discharges for each category during the same reporting period.

### 2.3. Data Analysis

The data analysis for this study involves assessing the technical efficiency and allocative efficiency of the hospitals. The following methods were used to analyze the data:

- Technical efficiency: was calculated by dividing the total number of hospital days for all patients for the same year by the total number of those patients excluding same-day cases and rehabilitation centers. This calculation provides an understanding of how efficiently hospitals are utilizing their capacity to treat patients.
- Allocative efficiency: was calculated by dividing the number of same-day cases (SDs) over the total number of discharges within the same year. The comparison was done across clusters based on the type and size of hospitals. The analysis also focuses on identifying potential waste in the system through the DOSA analysis and estimating the opportunity costs of having high DOSA rates in public hospitals. The required data were extracted from only one hospital that had a comprehensive patient-level data record, allowing tracking of the procedures conducted for patients. A total of 1060 patients who spent up to 10 days in the hospital before undergoing surgery without receiving any type of treatment were identified for the year 2019. We excluded those patients whose total length of stay after surgery was more than their DOSA, assuming their surgeries were not planned prior to their admission. The total DOSA for these patients was then calculated to assess the resulting cost of this inefficiency. The base cost or cost-per-bed per day was assumed based on information obtained from a clinical costing exercise conducted for that specific hospital.
- Productivity and complexity of care: was assessed using CMI, which is a statistical tool that can be used to assess the productivity and efficiency as well complexity of services. The CMI for a hospital during a specific period is calculated by dividing the sum of all DRG-relative weights by the number of patients. The CMI is then used by payers to adjust payment or reimbursement rates for hospitals. The CMI considers the total weighted episodes (WEs) instead of just the number of patients or reported episodes. Although the DRG weights available at the ABM portal are not specific to Saudi Arabia, they were used as a starting point since cost data was not available.

## 3. Results

### 3.1. Technical Efficiency

**Figure 1:**
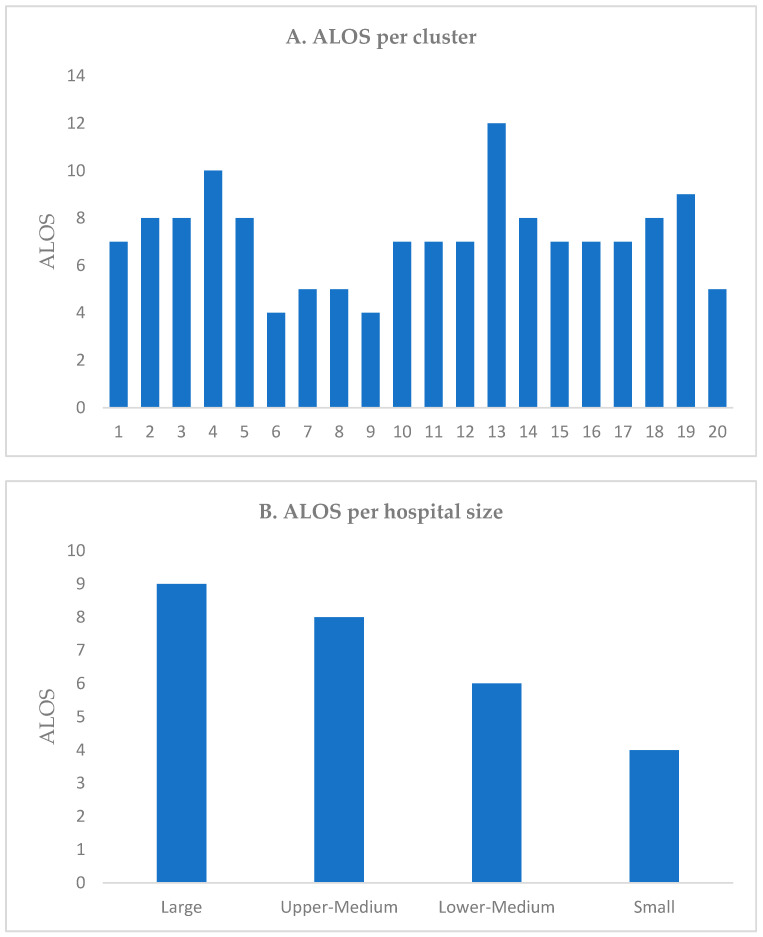
Average length of stay (ALOS) (**A**) per cluster and (**B**) hospital size.

We conducted an assessment of the cost of providing services to beneficiaries using the ALOS as a measure of technical efficiency regardless of how clinically appropriate those services are. Our analysis focused on the overall ALOS for all hospitals in 2019, excluding same-day cases and rehabilitation centers to ensure data accuracy. The findings revealed variations in ALOS across hospital clusters based on size and type. The overall ALOS for all hospitals in 2019 was 7 days. However, there were variations in ALOS based on hospital size and type. Large hospitals had the highest ALOS with an average of 9 days, while small hospitals had the lowest ALOS with an average of 4 days. Furthermore, ALOS exhibited wide variations across clusters, ranging from 4 to 12 days, depending on the type of hospital. Cluster 13 had the highest ALOS, while clusters 6 and 9 were the lowest. A comparison of ALOS across clusters at the aggregate level based on the hospital size and type is illustrated in Figure 1. Medical cities have a large LOS and specialty hospitals have the lower LOS (Appendix A).

### 3.2. Allocative Efficiency

#### 3.2.1. Same-Day Cases

**Figure 2:**
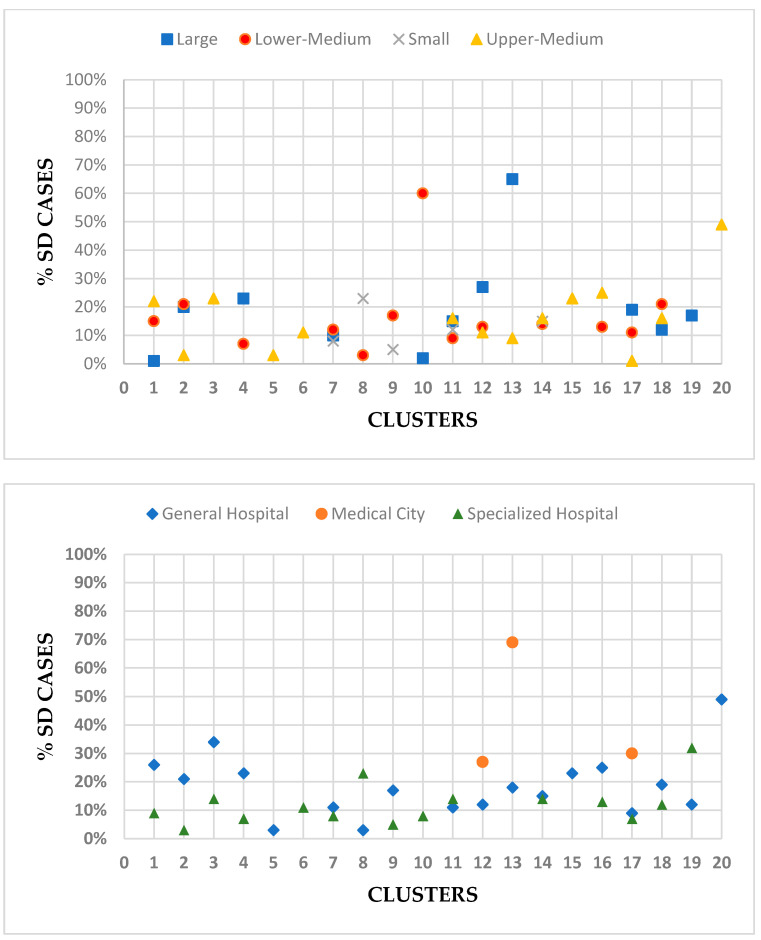
Percent of same-day cases per hospital size, type, and cluster.

Allocative efficiency measures whether patients were treated in an appropriate clinical manner. SD-episode percentage was highest for large hospitals with 19% of total episodes, while it was the lowest for small hospitals at 13% of total episodes. SD percentages were highest for large hospitals and medical cities, while specialized hospitals had the lowest SD percentages. Among clusters, cluster 13 had the highest average SD at 59%, while cluster 5 had the lowest average SD at 3%. Figure 2 provides the percentage of SD cases per cluster based on hospital size and type.

#### 3.2.2. Day of Surgery Attendance (DOSA) Analysis 

The day of surgery attendance (DOSA) statistics were analyzed to assess operational efficiency and identify potential waste in the system. Ideally, for non-complex cases, patients should be admitted on the same day as their surgery to reduce the length of stay (LOS) and waiting lists. By examining the number of patients occupying beds for multiple days before surgery, the study estimates potential cost savings of high DOSA rates. The potential cost savings for patients staying 2 to 5 days before surgery could amount to SAR 18 million, while patients staying 5 to 10 days before surgery could result in cost savings exceeding SAR 28 million. Table 2 provides comprehensive details regarding the potential cost savings across different scenarios and bed occupancy durations.

### 3.3. Productivity

**Figure 3:**
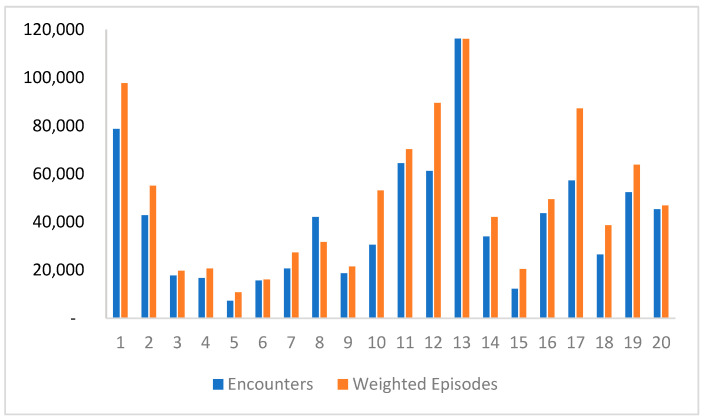
Weighted episodes vs. total encounters per cluster.

Productivity was assessed using CMI, which considers the total weighted episodes (WEs) instead of just the number of patients or reported episodes. Although the DRG weights available at the ABM portal are not specific to Saudi Arabia, they were used as a starting point since cost data were not available. Cluster 13 had the highest number of encounters and WEs, while cluster 5 had the lowest. Figure 3 illustrates the comparison between weighted episodes and total encounters per cluster. The MDCs and DRGs with the highest WEs were identified, indicating a higher resource consumption for treating those patients. MDCs with the highest number of weighted episodes is pregnancy, childbirth, and puerperium, followed closely by newborns and other neonates. When WEs are higher than encounters, it indicates that patients are consuming higher resources in order to treat them. Appendix B demonstrates WEs for each cluster broken down by MDC in 2019.

### 3.4. Case Mix Index

**Figure 4:**
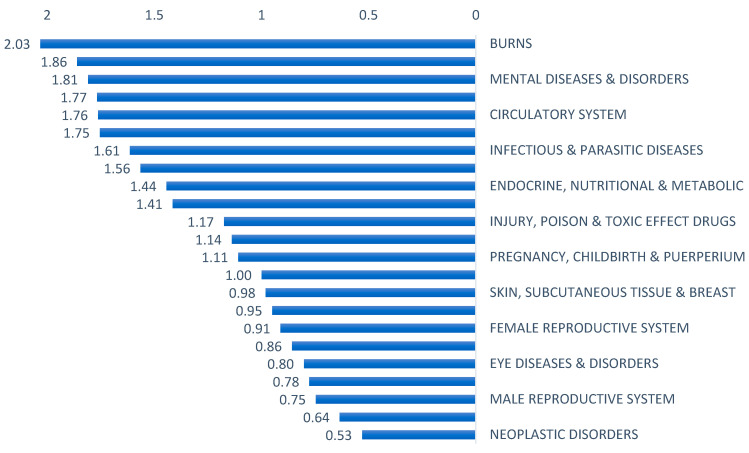
Case mix index by major diagnostic category, 2019.

We found that CMI varies across hospitals depending on the hospital size and type. The average CMI across all 67 hospitals was 1.26. At cluster level, the highest CMI was observed in 15 with CMI of 1.67, while clusters with lowest were 6 and 9 with CMI 1.02 for both clusters. Amongst provider types, medical cities had the highest average CMI at 1.47 followed by specialized hospitals and general hospitals at 1.32 and 1.21, respectively l (Table 3). Higher CMI for medical cities than specialized and general hospitals is consistent with the complexity and type of services utilized at these facilities, which may involve utilizing more resources, especially advanced technologies. One medical city MC1 with more than 92,000 encounters had an unexpectedly low CMI at 0.92, which is not representative of the expected case mix given the facility type. CMI also varied by the size of hospitals across clusters. Large hospitals had an average CMI of 1.42, while small hospitals had an average of CMI of 0.97. We also investigated CMI per MDC, with burn MDC having the highest CMI at 2.03 and neoplastic disorders MDC having the lowest CMI at 0.53 (Figure 4).

## 4. Discussion

This study assessed the efficiency of different MOH hospital in Saudi Arabia and enabled comparison across clusters. Our study, for the first time, reports on and measures efficiency using CMI in MOH hospitals. The CMI calculations allowed for determining how each hospital performed in terms of resources used and value produced and examined the consistency of CMI with hospital type based on the perceived type of services provided. Our findings reveal significant variations in technical and allocative efficiency across hospitals. 

Technical efficiency was assessed using ALOS and significant variation across hospitals was found, indicating differences in the complexity and types of services provided. Bed size has been shown to be positively related to ALOS. Knowing that small hospitals tend to treat less complicated cases, smaller hospitals generally exhibited shorter ALOS, suggesting efficiency and consistency with the type of services they usually provide compared to medium-sized and large hospitals. This is consistent with previous studies that have reported on small hospitals demonstrating higher efficiency compared to medium-sized and large hospitals [11,14]. Even though larger hospitals are expected to treat more complicated cases that require longer lengths of stay, these findings were consistent with another study that concluded that the longer ALOS for these hospitals is partially attributed to potential inefficiencies in patient flow and discharge processes [22,23]. Reducing ALOS is a priority for hospitals aiming to improve operations and reduce costs. In Saudi Arabia, 75.8% of assessed public hospitals were found to be technically inefficient. The average efficiency score was 0.76, indicating that these hospitals could have reduced their inputs by 24% without compromising the provision of health service [14]. Benchmarking against more efficient hospitals can help inefficient hospitals identify areas for improvement and implement best practices [24]. It worth noting that there is no single solution to address hospital inefficiency. Instead, a multi-faceted approach involving various interventions tailored to the hospital and its environment is essential. By considering multiple factors and adopting multi-intervention packages, hospitals can effectively tackle inefficiencies and enhance their overall performance [12].

Analyzing DOSA provides insights into operational efficiency. Ideally, patients should be admitted on the same day as their elective surgery to minimize length of stay and waiting times. However, our analysis revealed inefficiencies in the system, with patients experiencing prolonged hospital stays before their surgeries. Implementing a more streamlined admission policy and prioritizing DOSA admissions could result in substantial cost savings. Our analysis estimates a significant opportunity cost of up to SAR 11 million for patients who stayed in the hospital from 2 to 10 days before their surgeries. However, further ad-hoc analysis should be conducted to examine this issue in more details, considering the fact that some patients may require further preparation and stabilization prior to their elective surgeries. Comparing our findings to hospitals in the UK, we observe a significant disparity in DOSA. The UK is focusing on making this period shorter so patients’ LOSs could be shortened, allowing the hospital to receive more patients. Typically, patients should spend one night in the hospital before undergoing elective surgery. However, our analysis revealed that the average DOSA in the MOH hospital was 228 h, which is higher than UK hospitals [25,26]. This emphasizes the potential for improvement in reducing pre-surgery hospital stays and enhancing efficiency in the admission process. Improving DOSA can enhance operational efficiency, reduce costs, and enhance patient experiences. Strategies include enhanced scheduling, thorough preoperative assessments, pre-surgery checklists, dedicated pre-admission areas, streamlined discharge processes, improved communication, utilization of technology, and continuous monitoring [27,28]. These initiatives optimize resource utilization, minimize waiting times, and ensure a smoother surgical pathway for patients.

Allocative efficiency refers to the appropriate allocation of resources to ensure patients are treated in a clinically suitable manner. One way to assess allocative efficiency is by examining the percentage of same-day cases (SDs) out of total episodes. Variations in allocative efficiency were observed across hospitals, reflecting differences in clinical practices and case complexity. Large hospitals and medical cities had higher SD percentages, likely due to the advanced technologies and less intensive procedures. However, overall SDs were lower than those reported in Australia, whose SDs from their public hospital range from 43% to 54.3%. This mainly contributed to the implementation of case mix classifications. Monitoring activities and policy changes were implemented to keep track and stay ahead of regulatory requirements [29]. Standardized guidelines and evidence-based protocols can optimize resource allocation and enhance patient care [30]. Furthermore, implementing new provider payment methods can lead to cost savings and improved efficiency, which could be achieved through the new health reform in Saudi Arabia. The introduction and experimentation of DRG and other mixed provider-payment methods in China have shown advantages in cost reduction and reduced waiting times [31]. Additionally, the establishment of relevant laws and regulations is necessary to enable the differentiation of healthcare services provided. International evidence suggests that countries with gatekeepers, such as primary care physicians, tend to exhibit greater efficiency. However, it is essential to recognize that efficiency alone is not sufficient; doing the right thing is equally important to achieve optimal patient outcomes. Policymakers and healthcare providers must prioritize evidence-based decision making to ensure that interventions achieve desired health outcomes effectively. Muir Gray, an esteemed scholar in this field, has extensively emphasized the significance of aligning healthcare efforts with evidence and population needs. Value-based healthcare goes beyond efficiency, emphasizing outcomes and value for money. It delivers high-quality care that is effective and efficient, striking the right balance between cost-effectiveness and patient-centeredness. By adopting these principles, policymakers and healthcare providers can work towards achieving the best possible health outcomes for individuals and populations while making optimal use of available resources [10].

The analysis revealed variations in clusters productivity measured by CMI and weighted episodes (WEs). Cluster 13 exhibited the highest number of encounters and WEs, indicating higher resource consumption for patient treatment. Cluster 12 was more productive despite having a lower number of patients, suggesting efficient resource utilization. There was a substantial difference in CMIs among hospitals, ranging from 0.64 to 2.55. The hospital with the highest CMI indicates the utilization of extensive resources. This finding was further supported by the relatively longer average length of stay observed at this hospital. When considering the impact of hospital size on complexity of care, our analysis revealed that larger hospitals had a higher average CMI of 1.42 compared to smaller hospitals, which had an average CMI of 0.97. This suggests that larger hospitals often handle a greater variety of complex cases and require more extensive resource utilization. However, it is important to note that size alone does not solely determine the complexity of care, as other factors such as patient mix and available services also play a significant role. Hospitals with low CMI could benefit from benchmarking against similar peers to optimize resource utilization. Furthermore, the variation in CMIs across hospital types was noteworthy. Medical cities had the highest CMIs (1.47) due to specialized and advanced care services. Overall, the findings align with international benchmarks, but one medical city had an unexpectedly low CMI, potentially due to incomplete data submission or issues with clinical documentation and coding activities. Further analysis is needed to ensure the accuracy of CMI calculation by investigating the quality of clinical documentation and coding in MOH hospitals. 

Technical efficiency in a hospital is achieved when it maximizes outputs while utilizing a given level of inputs or resources, or conversely, when it minimizes inputs while producing the desired level and selection of outputs. The distinction between specialized and general hospitals has been a subject of investigation in efficiency studies. For example, a study analyzing various Italian hospitals discovered that specialized hospitals focusing on single treatments displayed higher transient inefficiency compared to general hospitals, suggesting potential economies of scope [32]. On the other hand, research conducted in three states of the USA using stochastic frontier cost functions indicated that general hospitals might be more efficient than specialized ones [33]. These findings highlight the importance of understanding the contextual factors that influence hospital efficiency and the need for tailored approaches to enhance overall performance. Internal hospital-level characteristics, such as ownership, size, specialization, teaching status, and membership in multihospital systems, impact efficiency. Additionally, factors like case mix and the ratio of outpatients to inpatients play a role. On the other hand, certain factors lie outside the hospital’s direct control, including geographic location, competition, and reimbursement systems [12].

The analysis reveals a similar pattern of variation in efficiency as found in a different country, emphasizing the importance of considering contextual factors and country-specific healthcare systems when assessing hospital efficiency. In Finland and Norway, the most efficient hospitals were observed to be small- and medium-sized local hospitals with a below-average DRG case mix. This suggests that efficiency is influenced by factors beyond hospital size and case mix, and further research is needed to understand the specific factors contributing to efficiency variations in different healthcare contexts [34,35]. Overall, CMI highlights the diversity in resource utilization and service intensity across hospitals. The presence of advanced technologies and a wider range of specialized services in medical cities could explain the higher CMIs observed in these facilities. Efficiency correlates with factors beyond technology, necessitating a comprehensive understanding of efficiency in regional hospitals [34]. It is important to interpret these findings cautiously, considering the limitations of CMI as a complexity measure. Further analysis, including patient profiles and resource allocation, is needed for a comprehensive understanding of complexity variations.

CMI provides valuable insights into a patient’s diagnosis, interventions, and treatment costs. The accuracy of CMI relies on proper clinical documentation, including additional diagnoses and comorbidities. High CMI may indicate accurate clinical documentation practices rather than just complex services [36]. Changes in case complexity and coding completeness can impact CMI. This indicates that most of the additional payments made to hospitals were justified by the increased complexity of patients hospitalized during that period [37]. Inefficiencies in healthcare systems include inadequate coordination between healthcare providers, fragmented care-delivery models, suboptimal use of technology and information systems, and variations in clinical decision-making processes. Targeting these inefficiencies improves resource allocation, reduces waste, and improves patient outcomes. A study assessed the impact of case mix index on postoperative outcomes of surgery for Medicare beneficiaries revealed that lower case mix index was associated with poorer outcomes, highlighting the importance of case complexity [38]. Implementation of the case-mix system has shown positive effects on clinical efficiency, with a decrease in technical inefficiency of 1.93 percent annually [39].

Efficiency in a health system cannot be fully captured by a single metric. Analyzing and understanding the root causes of inefficiencies is crucial for effective policymaking. To achieve this, it is essential to have standardized and detailed costing data, along with linked datasets and registries [11]. Two commonly utilized methods, DEA and SFA, are valuable tools to assess the efficiency and performance of hospitals and healthcare systems. By incorporating the case mix index (CMI), which considers the patient case complexity and severity, these analytical approaches offer a comprehensive framework to evaluate healthcare performance, identify best practices, and guide policy decisions. Monitoring the average CMI over time provides valuable insights into the changing patient population and resource needs. Comparing average CMI among hospitals helps identify differences in patient acuity and resource utilization, thereby facilitating benchmarking and performance evaluation and enabling hospitals to identify areas of improvement and learn from facilities with higher average CMIs. The average CMI also has reimbursement implications, as it affects payment models. A higher average CMI may indicate the need for increased financial support to address the resources required for complex cases. However, it’s essential to interpret average CMI alongside other metrics such as cost efficiency, quality outcomes, and patient satisfaction for a comprehensive understanding of hospital performance and informed decision making. Adopting a multi-dimensional reporting measurement system is necessary for fostering efficiency improvement, performance enhancement, and knowledge sharing [31]. Further analysis can investigate the quality of clinical documentation in MOH hospitals to ensure CMI accuracy. Additionally, periodic CMI assessment enables tracking of hospital activities to initiate more efficient and cost-effective practices while maintaining quality of care. 

The breakdown of WEs by major diagnostic categories (MDCs) provided further insights into the specific areas where resources are being consumed. Our study had interesting findings regarding the CMI across different MDCs. The burn MDC had the highest CMI (2.03), indicating higher case complexity within this category. Conversely, the neoplastic disorders MDC had the lowest CMI (0.53), suggesting relatively lower complexity. The observed lower CMI for neoplastic disorders can be attributed to the data submission for this MDC. In this case, the majority of reported cases were related to patients admitted as day cases specifically for receiving intravenous chemotherapy infusion. Since chemotherapy IV infusion typically involves less complex medical interventions and shorter lengths of stay, it leads to a lower overall CMI for the neoplastic MDC.

A study analyzed hospital output and resource consumption using patient-level data and compared the 20 most resource-consuming diagnosis related groups (DRGs) in Finland and Norway. It indicated that the most costly DRG was related to hip and knee replacements, while in Norway, it was associated with rehabilitation services. These findings illustrate variations in resource utilization and healthcare priorities between the two countries [34]. The consideration of various clinical indicators and patient outcomes, along with allocative efficiency and day-case rates, could be an area for future research. Incorporating these aspects into future research will contribute to advancing the understanding of healthcare efficiency and clinical appropriateness, ultimately leading to more effective and patient-centered healthcare delivery.

While this study provides insights on the efficiency of public hospitals in Saudi Arabia, there are important limitations to consider. One of the limitations of this analysis is the low number of encounters data “patient data” for several hospitals, which may compromise the accuracy of comparison. Future analysis could include a more detailed assessment of the average length of stay and number of patient encounters “multiple admissions” for specific services in each cluster. Furthermore, there are several other shortcomings that need to be addressed, such as how completeness of data for each encounter according to the minimum dataset MDS, like additional diagnoses and date and time of procedures. Another limitation in this study is that DOSA was only assessed in one hospital due to limited access to other hospital patient-level data. Additionally, exclusion criteria for DOSA investigation may require clinical judgment due to the differences in clinical practice for the different type of surgeries. This is because some surgeries may justify the prolonged length of stay prior to the surgery due to clinical evaluation or proper preparation while other surgeries do not. Further analysis could be conducted comparing DOSA based on patients’ procedure or the specialty of acute service they are receiving rather than DRG system, which doesn’t account for clinical differences across patients. Although the time frame of current study is small, it still gives an indication of hospital activity and allows for risk assessment to be performed. Another study’s limitations include the use of Australian cost weights that may not fully align with local considerations. The findings of our analysis were consistent with international benchmarks in terms of comparable CMI of hospitals/ clusters and contributes to informing policies for hospital operations and resource utilization in Saudi Arabia. 

## 5. Conclusions

This study sheds light on various aspects of hospital performance, including technical efficiency, allocative efficiency, and productivity. These findings emphasize the importance of optimizing hospital operations, reducing unnecessary bed occupancy, improving clinical practices, and enhancing resource allocation. The results can serve as a baseline for the different metrics to establish the national average that can be used to set targets for clusters and hospitals performance. It can also be used as the basis for further investigations and inform decision-making processes to improve the overall efficiency and effectiveness of healthcare delivery in the Kingdom of Saudi Arabia.

## Figures and Tables

**Table 1:** Characteristics of the hospitals included in this study.

Variables	*N* =67	%
Hospital type		
General Hospital *	42	63%
Specialized Hospital *	22	33%
Medical City *	3	4%
Hospital size		
Large (500 or more beds)	15	22%
Upper-Medium(300–499 beds)	20	30%
Lower-Medium (200–299 beds)	21	31%
Small (less than 200 beds)	11	16%

* Medical cities are large, advanced healthcare complexes located in major cities that offer comprehensive medical services and specialized treatments. They serve as major healthcare hubs, providing a wide range of medical departments and specialty centers; General hospitals are essential healthcare centers that offer a broad spectrum of medical services, including general medical care, emergency services, surgical procedures, and some specialized treatments. They are more widely distributed throughout the country; Specialty hospitals focus on specific medical fields and provide specialized care and treatments in areas such as cardiology, oncology, orthopedics, pediatrics, and more. They are equipped with specialized medical equipment and highly trained professionals in their respective fields.

**Table 2:** Opportunity cost of day of surgery admission (DOSA).

Days before Surgery	Number of Patents	Total DOSA	Cost of Bed Day (Scenario 1)	Opportunity Cost (Scenario 1)	Cost of Bed Day (Scenario 2)	Opportunity Cost (Scenario 2)
2	478	956	2000	1,912,000	3000	2,868,000
3	199	597	2000	1,194,000	3000	1,791,000
4	104	416	2000	832,000	3000	1,248,000
5	92	460	2000	920,000	3000	1,380,000
6	63	378	2000	756,000	3000	1,134,000
7	45	315	2000	630,000	3000	945,000
8	36	288	2000	576,000	3000	864,000
9	23	207	2000	414,000	3000	621,000
10	20	200	2000	400,000	3000	600,000
Total	1060	3817		7,634,000		11,451,000

DOSA 2: Patients staying two days in hospital before undergoing surgery. DOSA 3, 4, 5, 6, 7, 8, 9, and 10 denote days spent occupying a hospital bed before the surgery. Cost of bed scenarios are arbitrary assumptions. Monetary value is in SAR. Data are for the whole year of 2019. Data were extracted from same hospital.

**Table 3:** Case mix index per hospital size, type, and cluster.

Cluster	Hospital Size				Hospital Type			Average
	Large	Upper-Medium	Lower-Medium	Small	General Hospital	Medical City	Specialized Hospital	
1	2.05	1.18	1.19	-	1.19	-	1.6	1.35
2	1.41	1.29	1.15	-	1.28	-	1.29	1.28
3	-	1.11	-	-	1.06	-	1.16	1.11
4	1.25	-	1.2	-	1.25	-	1.2	1.23
5	-	1.47	-	-	1.47	-	-	1.47
6	-	1.02	-	-	-	-	1.02	1.02
7	1.32	-	1.34	0.73	1.33	-	0.73	1.13
8	-	-	1	1.11	1	-	1.11	1.04
9	-	-	1.15	0.76	1.15	-	0.76	1.02
10	1.83	-	0.95	-	-	-	1.39	1.39
11	1.17	1.4	1.3	0.88	0.99	-	1.42	1.1
12	1.65	1.12	1.6	-	1.24	1.65	2.55	1.53
13	1.15	1.22	-	-	1.3	0.92	-	1.18
14	-	1.4	1.28	1.08	1.24	-	1.28	1.26
15	-	1.67	-	-	1.67	-	-	1.67
16	-	1.14	1.16	-	1.14	-	1.16	1.14
17	1.48	1.6	1.29	1.7	1.53	1.83	1.31	1.51
18	1.4	1.91	1.13	-	1.52	-	1.4	1.48
19	1.22	-	-	-	1.22	-	1.23	1.22
20	-	1.07	-	-	1.07	-	-	1.07
Average	1.42	1.28	1.26	0.97	1.21	1.47	1.32	1.26

## Data Availability

The data presented in this study are available on request from the corresponding author.

## Appendix A

**Table A1:** Day cases ratio, ALOS, and Productivity per hospital.

Hospital Name	Cluster	Day Cases	Total Encounters	% Day-Case	ALOS	Weighted Episodes	CMI
1	1	50	7434	1%	7	15,257	2.05
2	1	2161	16,535	13%	5	20,348	1.23
3	1	843	5495	15%	7	6550	1.19
4	1	9187	24,026	38%	11	27,803	1.16
5	1	2885	24,384	12%	4	27,777	1.14
6	2	2137	10,544	20%	9	14,902	1.41
7	2	757	22,664	3%	8	29,197	1.29
8	2	2006	9563	21%	7	10,954	1.15
9	3	1306	9521	14%	8	11,035	1.16
10	3	2767	8175	34%	7	8702	1.06
11	4	2294	10,189	23%	15	12,760	1.25
12	4	454	6546	7%	5	7879	1.2
13	5	227	7321	3%	8	10,752	1.47
14	6	1767	15,694	11%	4	16,064	1.02
15	7	1612	13,329	12%	4	17,811	1.34
16	7	687	7075	10%	7	9347	1.32
17	7	21	259	8%	4	188	0.73
18	8	2064	8916	23%	6	9890	1.11
19	8	142	13,635	1%	3	13,872	1.02
20	9	415	7966	5%	4	7899	0.99
21	9	2438	11,083	22%	6	13,130	1.18
22	9	622	7334	8%	5	8140	1.11
23	9	12	261	5%	4	197	0.76
24	10	469	27,262	2%	11	49,984	1.83
25	10	1983	3313	60%	4	3158	0.95
26	11	2	73	3%	12	127	1.73
27	11	703	3562	20%	7	5164	1.45
28	11	22	121	18%	2	165	1.37
29	11	382	3081	12%	4	4180	1.36
30	11	1840	12,417	15%	4	14,583	1.17
31	11	1801	17,920	10%	25	20,530	1.15
32	11	1212	15,776	8%	4	15,913	1.01
33	11	103	1096	9%	3	983	0.9
34	11	1115	8845	13%	3	7573	0.86
35	11	15	86	17%	4	68	0.79
36	11	4	115	3%	2	84	0.73
37	11	191	1397	14%	2	890	0.64
38	12	-	6	0%	1576	15	2.55
39	12	9226	33,993	27%	7	56,029	1.65
40	12	517	4988	10%	10	7061	1.42
41	12	1013	8209	12%	11	10,210	1.24
42	12	1116	7282	15%	8	8654	1.19
43	12	745	6780	11%	5	7566	1.12
44	13	3170	11,152	28%	37	15,442	1.38
45	13	1164	12,452	9%	7	15,185	1.22
46	13	63,832	92,691	69%	12	85,514	0.92
47	14	16	98	16%	12	137	1.4
48	14	1912	14,579	13%	8	20,155	1.38
49	14	1405	9602	15%	4	11,295	1.18
50	14	1440	9731	15%	4	10,524	1.08
51	15	2791	12,249	23%	7	20,485	1.67
52	16	436	10,732	4%	11	13,397	1.25
53	16	2201	17,314	13%	5	20,097	1.16
54	16	6147	15,560	40%	7	15,963	1.03
55	17	7399	24,913	30%	12	45,625	1.83
56	17	-	20	0%	332	34	1.7
57	17	13	1166	1%	4	1860	1.6
58	17	855	4932	17%	9	6787	1.38
59	17	790	7300	11%	9	9423	1.29
60	17	846	18,959	4%	3	23,477	1.24
61	18	1462	8889	16%	15	16,940	1.91
62	18	797	6715	12%	5	9381	1.4
63	18	2304	10,913	21%	5	12,345	1.13
64	19	4198	13,251	32%	17	16,316	1.23
65	19	4540	39,127	12%	7	47,550	1.22
66	20	1444	15,342	9%	6	18,123	1.18
67	20	20,939	30,006	70%	4	28,706	0.96

## Appendix B

**Table A2:** Comparison of Major Diagnosis Categories (MDC) across cluster.

MDC Description	1	2	3	4	4	5	7	8	9	10	11	12	13	14	15	16	17	18	19	20	Total
Pregnancy, Childbirth & Puerperium	14,525	14,183	3924	2550	5565	2132	6781	10,778	2757	2013	18,693	10,658	14,286	16,954	3927	14,967	11,702	7514	14,247	8079	186,231
Newborns & Other Neonates	17,959	11,929	3892	3849	5409	2234	4962	8658	1909	5272	13,529	12,107	12,191	9030	5571	6173	13,953	4869	13,862	10,025	167,384
Musculoskeletal Sys & Conn Tissue	8555	4637	1538	2543	43	1143	2450	923	2323	3950	4525	12,096	8615	2028	1897	3802	4214	4207	4548	3063	77,101
Circulatory System	2199	1811	591	1536	24	412	1093	583	1409	7950	3158	6673	11,566	441	338	3655	19,767	884	2666	2700	69,453
Respiratory System	7673	3484	1781	1365	2278	809	2061	2659	2375	4567	5330	6538	6981	2525	1080	2641	5063	3072	3000	3724	69,004
Nervous System	6225	2888	1025	1726	261	1130	2132	1168	2148	3915	3693	9416	10,283	1524	1826	2466	5004	3562	3736	3360	67,488
Digestive System	6841	2988	1499	976	728	437	1652	1703	1847	3172	4861	6084	7674	1830	729	3437	5321	2154	3937	2722	60,593
Endocrine, Nutritional & Metabolic	2903	1958	1115	1052	81	345	731	567	799	2392	2601	4401	3488	631	472	1417	3518	1340	2121	1958	33,888
Kidney & Urinary Tract	4971	1562	494	922	138	260	1007	453	814	3167	1708	2681	3491	630	857	1191	2922	1598	1510	2760	33,138
Hepatobiliary System & Pancreas	3921	1391	556	715	11	216	904	397	1083	3450	2328	3232	2916	464	448	1259	2867	1595	2425	1871	32,050
Neoplastic Disorders	1356	191	63	127	20	6	89	45	35	4031	736	925	20,339	50	15	612	1779	375	110	57	30,962
Ear, Nose, Mouth & Throat	3133	644	622	862	899	127	703	992	1046	1310	2971	3719	2640	2038	456	1485	1969	1728	1840	1342	30,525
Blood, Blood Form Organs, Immunology	5557	2266	291	687	116	113	376	324	402	1967	805	1530	2936	702	212	531	1250	508	513	1238	22,327
Skin, Subcutaneous Tissue & Breast	3369	1765	451	390	50	123	518	459	378	1199	1391	1745	2082	295	228	982	1750	834	1585	875	20,469
Eye Diseases & Disorders	1244	52	676	17	5	2	314	156	718	1938	653	823	461	628	534	1448	1784	1746	4486	616	18,299
Injury, Poison & Toxic Effect Drugs	1195	788	540	295	101	341	779	599	693	346	997	2546	1102	566	704	1404	595	1271	1344	874	17,078
Infectious & Parasitic Diseases	2008	967	191	306	123	265	204	546	275	1125	776	1094	1355	350	363	428	1565	395	475	605	13,417
Female Reproductive System	1179	537	126	92	78	137	205	184	150	449	558	745	1536	666	218	356	1181	273	595	407	9671
Male Reproductive System	1551	435	174	138	83	49	245	202	195	204	443	796	574	575	131	413	493	412	483	233	7828
Factors Influencing Health Status	410	236	43	429	34	176	60	158	69	634	129	452	1355	81	39	499	271	94	118	131	5417
Burns	676	267	78	24	-	283	58	37	18	10	90	1158	116	33	434	234	184	169	186	136	4189
Mental Diseases & Disorders	260	68	63	34	15	10	19	70	21	73	226	102	147	69	6	48	57	68	68	49	1471
Alcohol/Drug Use Disorders	26	4	5	3	1	3	2	2	5	7	59	14	8	2	-	9	-	-	11	6	166
Total	97,736	55,053	19,737	20,639	16,064	10,752	27,346	31,662	21,467	53,141	70,260	89,535	116,141	42,111	20,485	49,456	87,206	38,666	63,866	46,829	978,151
